# Engineering application of vacuum preloading combined with electroosmosis technique in excavation of soft soil on complex terrain

**DOI:** 10.1371/journal.pone.0288026

**Published:** 2023-06-29

**Authors:** Zhaohua Sun, Jingxian Geng, Guowei Wei, Wenjin Li

**Affiliations:** 1 School of Transportation and Civil Engineering, Nantong University, Nantong, China; 2 Key Laboratory of New Technology for Construction of Cities in Mountain Area, Ministry of Education, Chongqing University, Chongqing, China; 3 Jiangsu Zhongnan Construction Industry Group Co. LTD, Haimen, China; University of Sulaimani, IRAQ

## Abstract

This paper presents the design and construction of vacuum preloading incorporated with electroosmosis (VPE) engineering application for the treatment of soft soil on complex terrain for sluice foundation excavation in order to reduce the amount of cement used in construction. Monitoring was conducted during the VPE treatment and laboratory geotechnical tests were carried out once the treatment came to an end. Results show that the electrification mode has a significant influence on electric energy consumption. Stepped-up voltage helped in saving electric energy while electrode conversion consumed a lot of electric energy. The dispersion of soil parameters became larger after VPE treatment. The stability of physical parameters is better than the mechanical parameters, and the latter is better than the deformation parameters. Soil water content has a linear relationship with density and compression coefficient. The given linear fitting equations can help in simplifying the calculation and acquisition of these indexes. Although the mean values of the soil index parameters slightly improved, their coefficient of variation (COV) significantly increased. These locations with improved index parameters scattering in the construction site ensured that the subsequent construction tasks such as pit slope and excavation were successfully realized in this area.

## Introduction

Due to the rapid economic development and reform of social policies, construction activities of civil and hydraulic underground structures have been increasing significantly. Soft soil with properties such as high liquidity, high water content, and low permeability often emerged in the process of foundation construction, hence presenting improvement or excavation challenges to the engineers [1–3]. Expand cooperation on new energy, energy conservation, and environmental protection has been a hot topic about engineering construction in soft soil foundations [4, 5]. In soft soil areas, during the process of foundation pit excavation soil below the excavation surface is subject to obvious vertical unloading, leading inevitably to rebound deformation of soil [6]. An increase in excavation depth, results in the difference in heights between the ground surface and bottom of the pit growing larger. Unexpected transmissive layers within the low hydraulic conductivity materials may lead to instability at the bottom of the excavation [7]. Stabilization of the foundation pit slope is one of the key problems during excavation. Strengthening of soft soil by means of ground improvement has proven to be a useful technique for the stabilization of the excavation and the control of unloading deformation [8].

In soft soil area, the soft soil usually needs to be reinforced before excavation of foundation pit. In foundation pit engineering the selection and application of soft soil reinforcement technology should be considered according to engineering geological conditions, construction conditions, design of foundation pit excavation, and other environmental and economic requirements [9–11]. At present, there are several reinforcement techniques for the excavated soft soil strengthening such as grouting [9], high pressure jet grouting [12], deep mixing [13], and dewatering [14] with different application conditions, technological characteristics, and economic suitability. The former three methods are accomplished by pumping a cement-based grout into the soil or mixing with the soil forming soil-cement column. The use of cement-soil mixing piles for providing stability of soft or loose soils also has yet widely spread in the excavation site [15]. Hu [16] presented the application of cement-soil mix piles to a deep excavation in soft soils adjacent to the Shanghai Metro tunnels. Although these technologies have recently further been applied in settlement control of soft soils, slope stabilization, and the formation of composite gravity structures, there are still major environmental concerns with cement production such as various gas emissions and energy consumptions [17]. According to the application practice of soft soil reinforcement in foundation pit excavation, Shi [18] classified the design form and layout of soft soil reinforcement, and pointed out that reasonable combination and flexible application should be made according to the problems to be solved.

Pumping consolidation or precompression by pumping water out of the ground was used to reduce the amount of water in soft soil layers and to improve the soil shear strength and deformation modulus before excavation [16]. The dewatering methods used in excavation engineering usually include pumping wells, light well points, ejector well points, electroosmotic well points and etc. [19]. The vacuum preloading method is also used to strengthen soft soil in excavation engineering, after four months of treatment the improved ground can be excavated with ease [20]. Pujades et al. [21] highlighted the importance of soil characterization for selecting the most efficient dewatering method when excavating under the water table. However, these dewatering methods were barely satisfactory to ensure stable conditions or with a long construction period when the excavation is undertaken in a low hydraulic conductivity soil. Through some laboratory and in-situ tests vacuum preloading combined with the electroosmosis method (VPE) has been proven to be suitable for low permeability soft clay strengthening with faster construction time [22–24]. However, the actual engineering application of VPE has not been reported.

In this paper, an in-situ application of VPE in the excavation engineering of a complex foundation pit in the tidal flat area is presented. The reinforcement effect of VPE on soft soil within the excavation range of the foundation pit is evaluated by monitoring the changes in current, vacuum pressure, surface settlement, and pore water pressure in soil on site, as well as the results of the indoor soil test. Based on these evaluations, some suggestions are developed to help engineers better design the VPE process and reasonably evaluate the strengthening effect.

## Project overview

**Fig 1:**
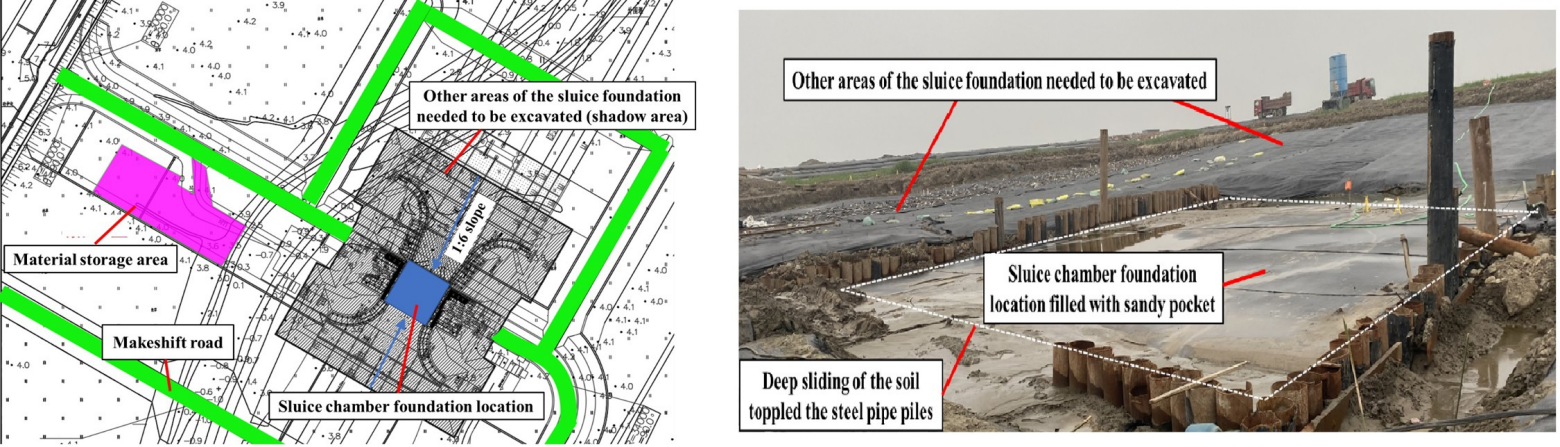
Current situation of the sluice foundation pit excavation project. a) Layout of the sluice foundation pit. b) Deep sliding occurred in the soil which damaged part of the pile foundation in the sluice chamber, when the site experienced excavation by using tube well, light well point, and open ditch dewatering technologies.

A sluice foundation pit excavation project was located on Chongming Dao, Yangtze estuary north of Shanghai, China. The design excavation depth ranged from 4.25–6.90 m, and covers an area of 12000 m^2^. On site, the pile foundation construction of the main structure of the sluice was completed first, mainly using pre-stressed high-strength concrete (PHC) pipe piles, triaxial and biaxial cement mixing piles. Then cement mixing piles were installed around the foundation pit and used as a water-proof curtain during dewatering and excavation. According to the original plan, tube well, light well point, and open ditch dewatering technologies were adopted in the foundation pit. However, the completion of the wells in soft soil was difficult due to borehole collapsing, which significantly increased the smearing and well resistance effect and decreased the water collection performance and drainage effect. Therefore, in the process of mechanical excavation, it was found that the soil moisture content was still high with a plastic fluid behaviour and low bearing capacity making it difficult for excavating machines to enter the field. Initially, after fifteen days of drainage, the foundation pit excavation was to start with a slope ratio of 1:3 but due to the challenges aforementioned the actual excavation slope ratio was decreased to 1:6 and deep sliding still occurred in the soil which damaged part of the pile foundation in the sluice chamber and empty container location, as shown in Fig 1, meaning the excavation had to be suspended. In view of the excavation disturbance on beach soft soil on complex terrain, it is urgent to find a suitable treatment method in an effort to promote the rapid drainage and strength of the soil, so as to meet the requirements of slope excavation construction.

### Geological and hydrological conditions

According to the geological survey records, the current distribution of the soil within the excavation depth was mainly mucky silty clay, and some mucky clay distributed in the shallow excavation depth area. Two soil layers were all recently deposited tidal flat with high water content, compressibility, and void ratio in a fluid plastic state. Mucky clay contains organic matter and humus with a layer thickness of 0.8–2.1 m, while mucky silty clay contains mica and organic matter with a layer thickness of 0.6–16.1 m.

The shallow groundwater of the site belongs to pore phreatic water, which mainly exists in the mucky clay layer, mucky silty clay layer and its adjacent soil layer. It is near the Yangtze River estuary and is directly fed by the river water. The water level dynamics are mainly controlled by the atmospheric precipitation and tide etc.

### Physical and mechanical properties of soil

The physical, mechanical, and deformation parameters of the soil were obtained by laboratory tests based on the canonical standard ASTM. Table 1 summarizes the available data on the inherent variability of some common index parameters. COV is the coefficient of variation of inherent variability, which is equal to the standard deviation of the inherent soil variability divided by the mean soil property trend. Generally, the greater the COV, the greater of the data dispersion degree. Conversely, the smaller the degree of data dispersion, the better the stability. The COV of inherent variability for the permeability coefficient and particle size distribution is larger than 25%, that for the liquidity index, cohesion, compression coefficient, and compression modulus are ranging from 10% to 15%, while that for other parameters are below 10%. Particle size is one of the main factors influences the soil permeability, the larger variability of the soil particle size determined the larger variability of the permeability coefficient. Although the soil water content is not particularly high in the range of 36% to 47%, its liquidity index is larger than 1 indicating that the soil is in a flow-plastic state. The particle grain sizes are all in the range of 0.005–0.075 mm, which means that the soil constituent is silt. The soil samples were classified as mucky silty clay according to the void ratio between 1 to 1.5, with water content larger than the liquid limit, and a plastic index between 10–17.

**Table 1:** Inherent variability of the index parameters before treatment.

Property		No. of data	Property value		Property COV (%)
			Range	Mean	
**Water content (%)**		95	36–47	42	6
**Density (g/m** ^**3**^ **)**		95	1.74–1.84	1.77	1
**Void ratio**		95	1.03–1.29	1.20	6
**Liquid limit (%)**		95	34–39	37	3
**Plastic limit (%)**		95	19–23	21	5
**Liquidity index**		95	1–2	1.4	13
**Plastic index**		95	15–17	16	4
**Permeability coefficient (cm/s)**		95	3.14 × 10^−7^-	6.29ⅹ10^−7^	27
			1.06 × 10^−6^		
**Cohesion (kPa)**		89	10–17	12	13
**Internal friction angle (** ^**0**^ **)**		89	16–19	17	5
**Compression coefficient (MPa** ^**-1**^ **)**		67	0.49–0.95	0.76	15
**Compression modulus (MPa)**		67	2.4–4.1	2.93	14
**Particle size analysis**	**0.075–0.05 mm (%)**	37	4–21.1	12.6	38
	**0.05–0.01 mm (%)**		22–73.8	53.6	25
	**0.01–0.005 mm (%)**		2.1–8.1	4.4	33

### Schemes comparison

Combined with the soil characteristics, construction requirements, and current situation of the project, soft soil foundation treatment methods were considered, such as in situ solidification, vacuum preloading, and vacuum preloading incorporated with electroosmosis. The construction craft, scope of application, and technical and economical characteristics of the three methods were comprehensively analyzed. In situ solidification method has a short construction period and good reinforcement effect with 14–21 $/m^3^, but the excavated solidified soil would become abandoned soil causing a waste of resources and the addition of cement in the soil increased the amount of earthwork. The vacuum preloading method is a mature technique with a low cost of 11–14 $/m^2^, but its construction period is long [25], which affects the project progress. According to present in-situ tests [24], VPE is suitable for the treatment of soft soil with high water content and low permeability with 22–42 $/m^2^, more crucially its construction period is short. Further technical explorations need to be carried out for the application of VPE in large area field engineering. Finally, VPE was chosen to treat the soft soil which was to be excavated due to its less cost and short duration.

### Design and construction of VPE

**Fig 2:**
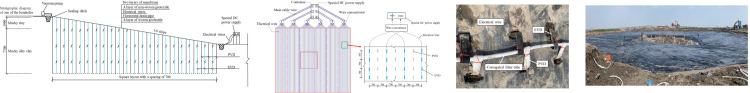
Implementation of VPE used in the excavation area with complex terrain. a) Cross-section scheme. b) Wiring diagram and layout plan of EVDs and PVDs. c) Connection of PVDs and EVDs with corrugated filter tube. d) Real scene of the construction site covered by vacuum membrane (unit mm).

Fig 2 illustrates the design scheme of VPE used in the excavation area with complex terrain. The construction site with a sloping surface first went through the clearing of debris and an engineering survey. Moreover, the slope was trimmed into step shape to facilitate the operation of a crawler-type plug-in machine, which was used to insert the vertical drains and electrodes into the soil. Since the bearing capacity of the disturbed soil was too low making it difficult for the machines or personnel to operate, a layer of non-woven geotextile was laid on the soil surface to achieve a better bearing capacity permitting personnel to work on the top. Plate-shaped plastic vertical drains (PVDs) and electric vertical drains (EVDs) were vertically inserted into the excavation depth of the soil with a spacing of 0.7 m in a square layout, as shown in Fig 2A and 2B. Detailed information for EVDs has been introduced by Sun et al. [26]. Above the soil surface 1 m of PVDs and EVDs were reserved and wound around the horizontal corrugated filter tube (40 mm in diameter) with a spacing of 1.4 m, as shown in Fig 2C.

**Fig 3:**
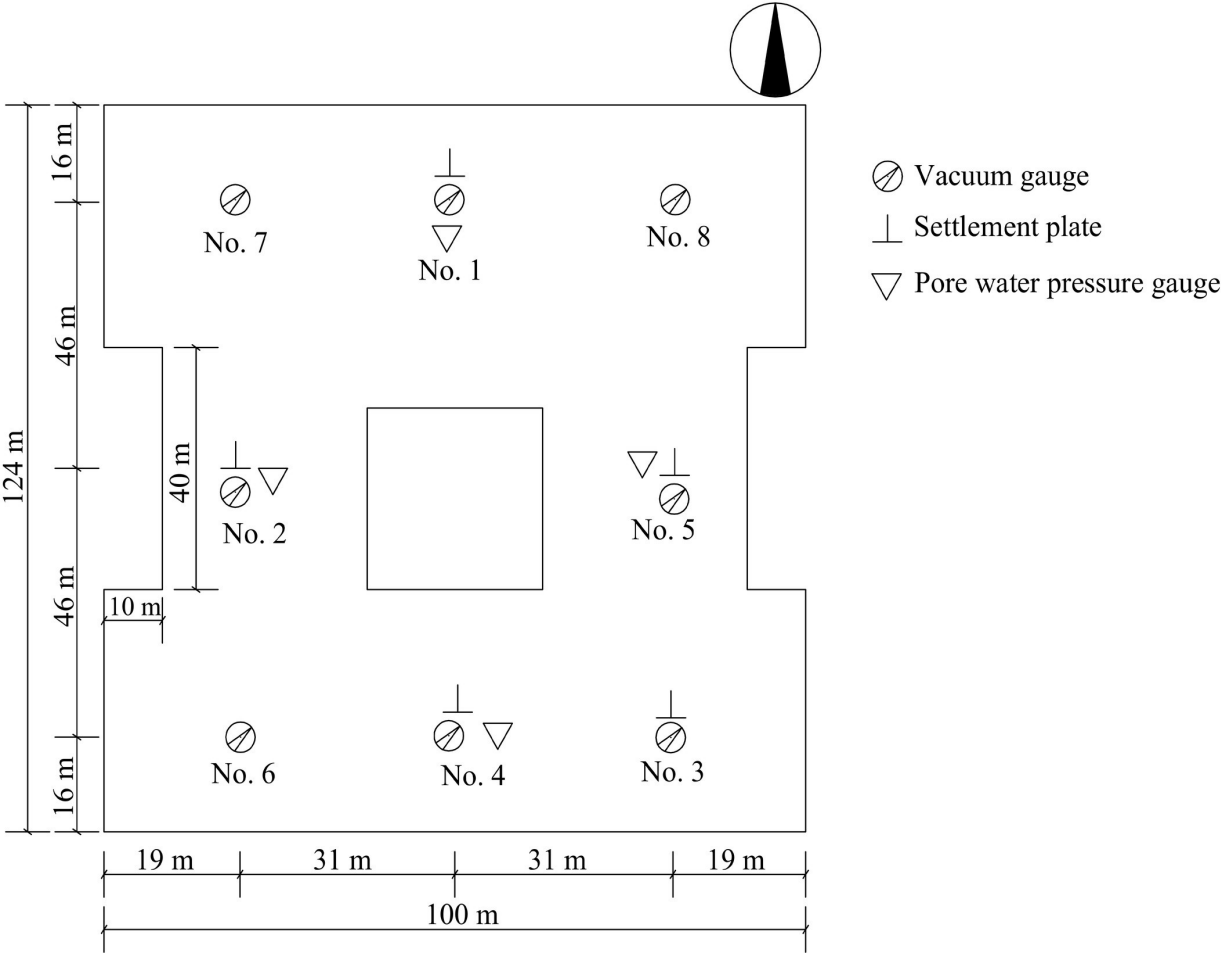
Layout of the monitoring point for vacuum pressure, surface settlement, and pore water pressure.

There are six wire concentrators connected to the EVDs on-site to the special direct-current (DC) power supply by electrical wires and main cable wires. The wiring diagram was illustrated in Fig 2B. Each wire concentrator is connected to 14–18 electrical wires, and each electrical wire is connected to 108–150 EVDs. The special DC power has high-voltage parameters and automatic control function according to the settlings. After that a layer of woven geotextile and two layers of vacuum membrane were placed on site. The edge of the membrane was embedded in the sealing ditch 1 m deep. In order to monitor the vacuum degree, pore water pressure, and surface settlement of the construction site, there were eight vacuum gauges, twelve pore water pressure gauges buried in four locations at depths of 1 m, 2 m, and 3 m, and five settlement plates were evenly distributed on site in the north, south, east, west and southeast directions, as shown in Fig 3. A 7.5 kW vacuum pump was deployed every 1000 m^2^. Other processes were similar to the conventional vacuum preloading method. Real scene of the construction site covered by vacuum membranes was illustrated in Fig 2D.

The combination mode of vacuum preloading and electroosmosis was divided into two stages, that were vacuum preloading first and then vacuum preloading and electroosmosis jointly reinforcement. The vacuum pumps started working on 2 June 2021 (first day), while the special DC power supply working time was 28 June 2021(27 th day). After that they continuous went on until the end of the reinforcement on 22 July 2021 (51 st day). When the pore water pressure dissipated obviously, the surface settlement curves converged, and the reinforcement came to an end. After the reinforcement, in-situ drilling and sampling were carried out for geotechnical laboratory testing, such as water content, density, Atterberg limits, shear strength index, compressibility coefficient and etc.

## Results and analysis

### Vacuum pressure

The sealing of the excavation area with complex terrain was hard work. The vacuum pressure under the membrane increased to 70 kPa after the jet vacuum pumps sucking lasted for almost seven days. Subsequently, the vacuum degree under the membrane of the eight measuring points fluctuated in the range of 70 to 82 kPa. Due to the influence of typhoon “fireworks” on July 20, 2021, the vacuum degree under the membrane decreased continuously and could only maintain 60 kPa. The vacuum pressure at PVDs and EVDs in the soil was obviously lower than that under the membrane [27].

### Electric current and electric energy consumption of electroosmosis

**Fig 4:**
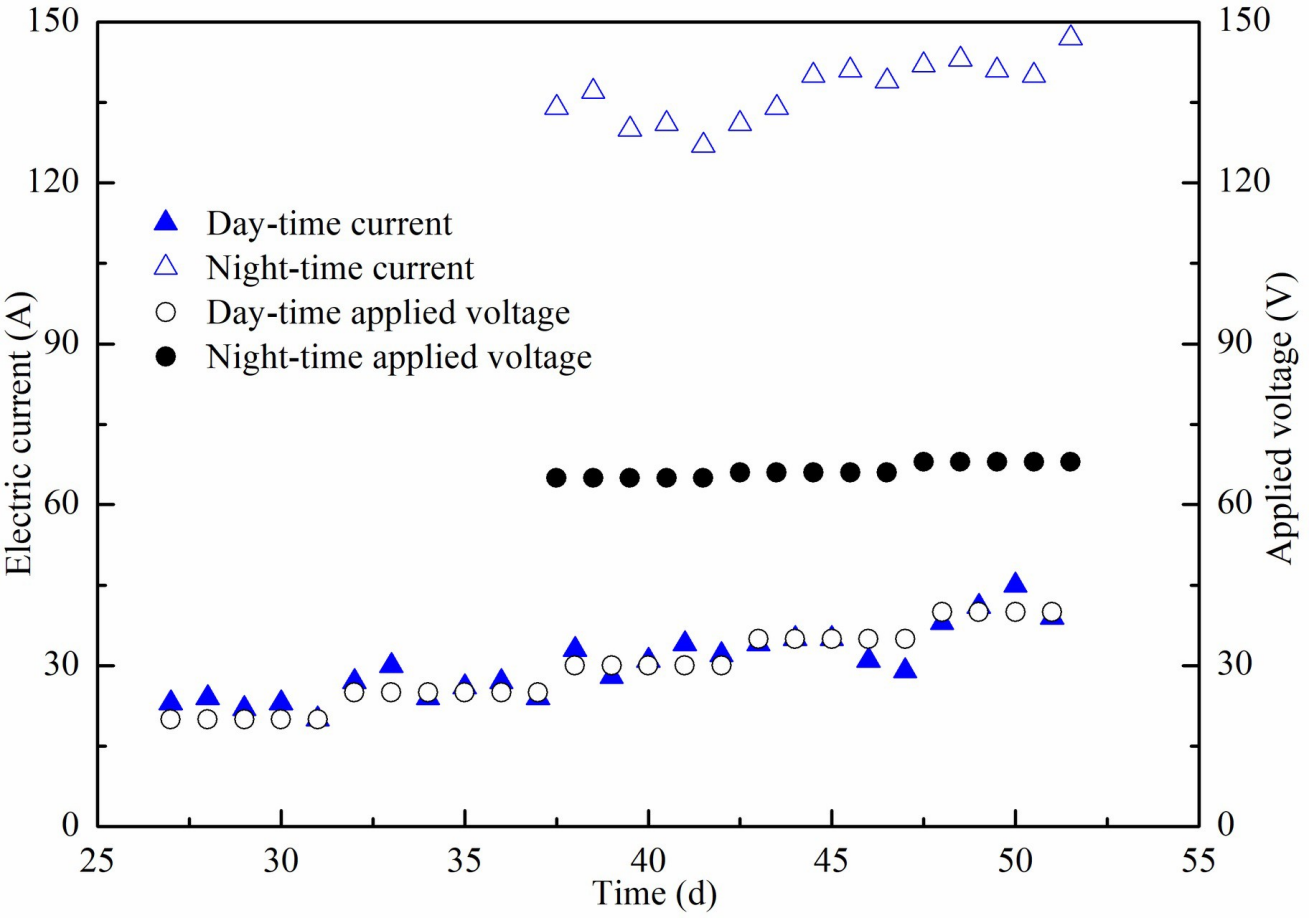
Electric current variation under different applied voltage of NO.1 special DC power supply.

**Fig 5:**
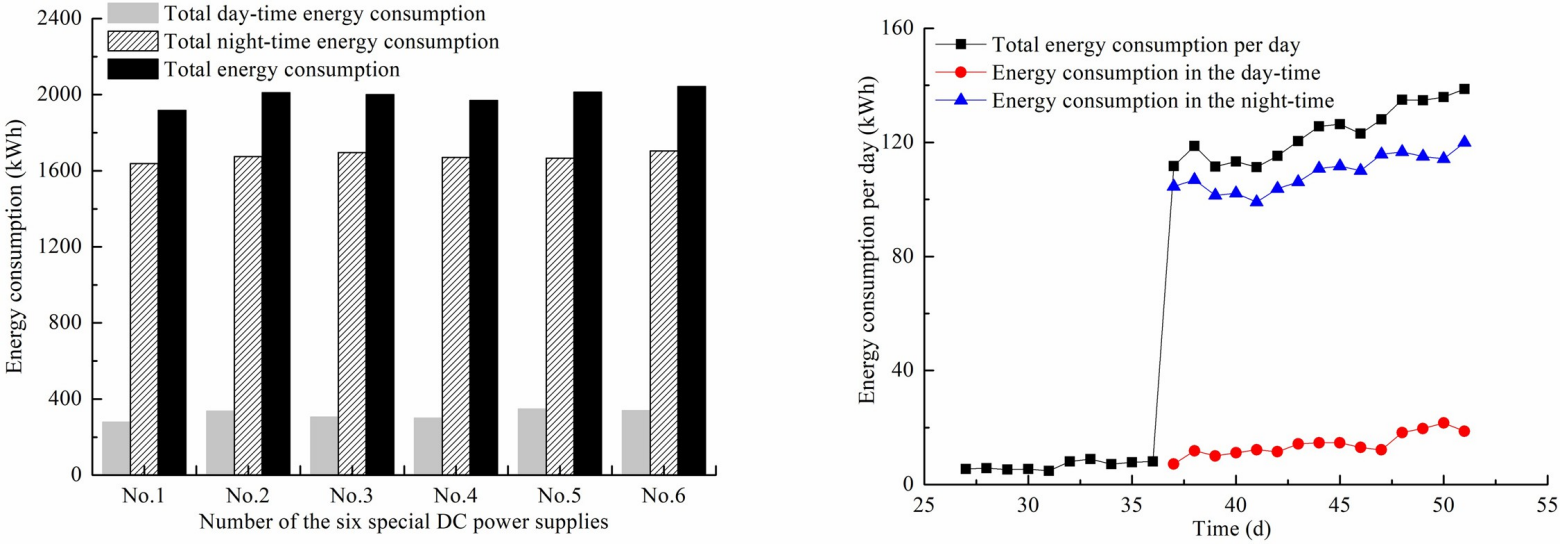
Electric energy consumption. a) Electric energy consumption of six special DC power supplies. b) Electric energy consumption per day for No. 1 special DC power.

The stepped-up steady voltage mode and electrode conversion technology were adopted in the field. The electric current variation trend was similar for six special DC power supplies, so only one of the special DC power supplies NO.1 displayed electric current variation under different applied voltage presented here, as shown in Fig 4. On the 27th day of vacuum preloading reinforcement, a steady voltage was applied to the EVDs during the day from 7 am to 6 pm and the initial voltage was 20 V, which was increased by 5 V every five days. From the 37th day, day-time applied voltage following the previous mode, while higher voltage was applied and the polarity of the electrodes was reversed in the night from 6 pm to 7 am. The values of during day electric current ranged from 20 A to 45 A which were close to that of its corresponding applied voltages. When the voltage was adjusted to about 65 V and reversed the polarity of the electrodes in the night, the electric current reached higher than 130 A. Reversing the electrodes at night was to generate reversed electroosmotic flow, which was conducive to reduce the interface resistance between the anodes and the soil. The stepped-up voltage during the day and electrode conversion during the night were helpful in maintaining the electric current in the soil during the day and it did not decrease but was rising slowly. According to the above electrification method, the total electric energy consumption for each special DC power supply was close, which was the sum of day and night electric energy consumption, as shown in Fig 5A. The electrification mode during the day did not consume too much electrical energy and its total values not more than 400 kWh, while the electric energy consumption during the night was striking (S1 and S2 Datasets).

Fig 5B provides an example of electric energy consumption per day for No. 1 special DC power. The electric energy consumption per day between days 27 to 36 (only during the day) was lower than 10 kWh, while after that the total electric energy consumption per day varied between 110 and 140 kWh. The electric energy consumption during the night per day from the 37th day accounted for more than 85% of the total energy consumption per day. The higher voltage applied and electrode conversion during the night consumed a large amount of electric energy. The lower current and voltage applied during the day did not consume much electric energy. Hence, the electrification mode has a significant influence on electric energy consumption.

### Pore water pressure

**Fig 6:**
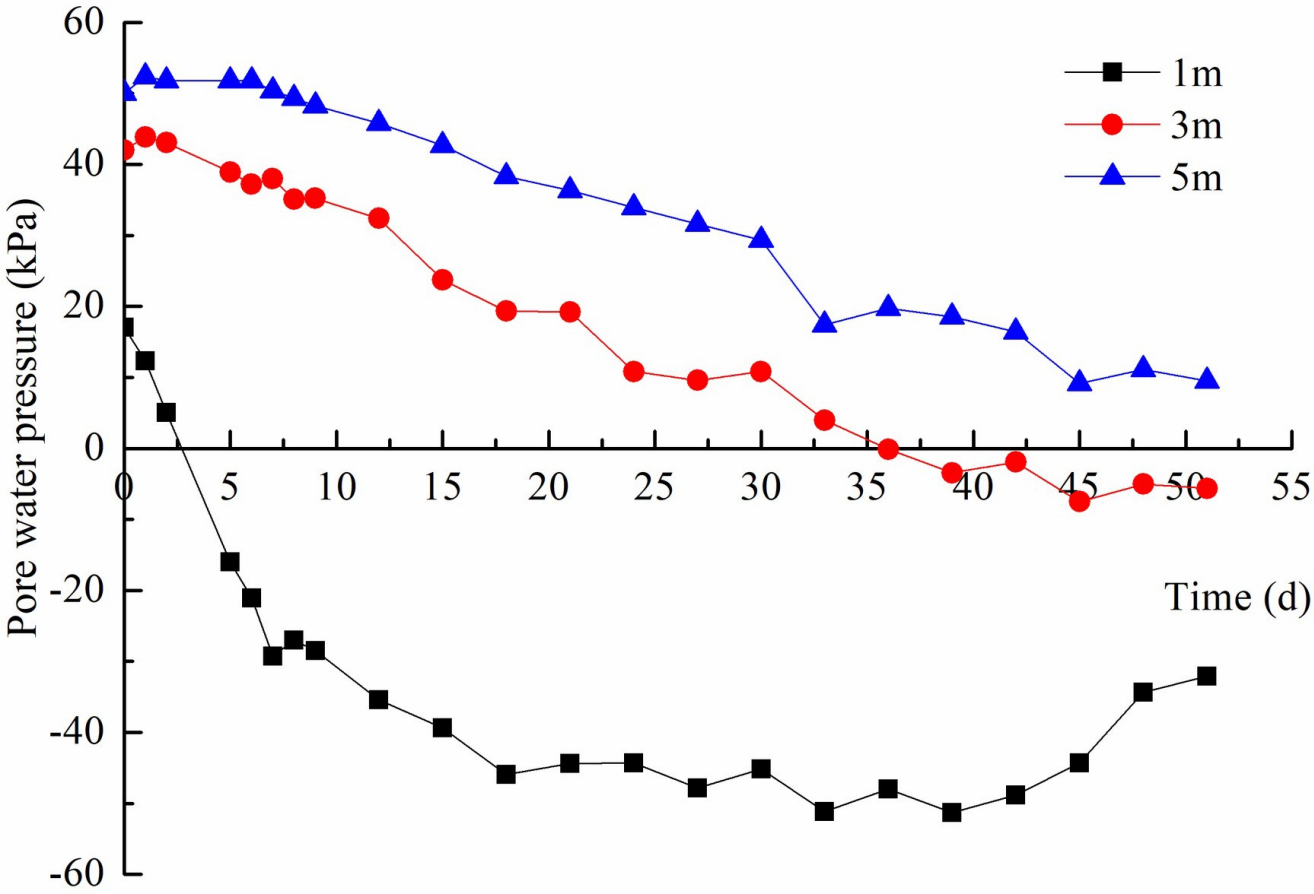
Pore water pressure variation.

The dissipation of pore water pressure decreased with increasing depth, which was mainly because the vacuum degree decayed with increasing depth. Taking one of the measuring points as an example, the variation of pore water pressure at 1 m, 3 m, and 5 m was introduced, as shown in Fig 6. The pore water pressure at 1 m is greatly affected by the vacuum degree, and decreases rapidly to a negative value. Its maximum dissipation value was about 68 kPa. The dissipation rate of pore water pressure at 3 m and 5 m was close and dissipated to 48 kPa and 40 kPa, respectively. In the later period, these pore water pressures rebounded especially at 1 m depth due to the decrease of vacuum degree (S3 Dataset).

### Surface settlement

**Fig 7:**
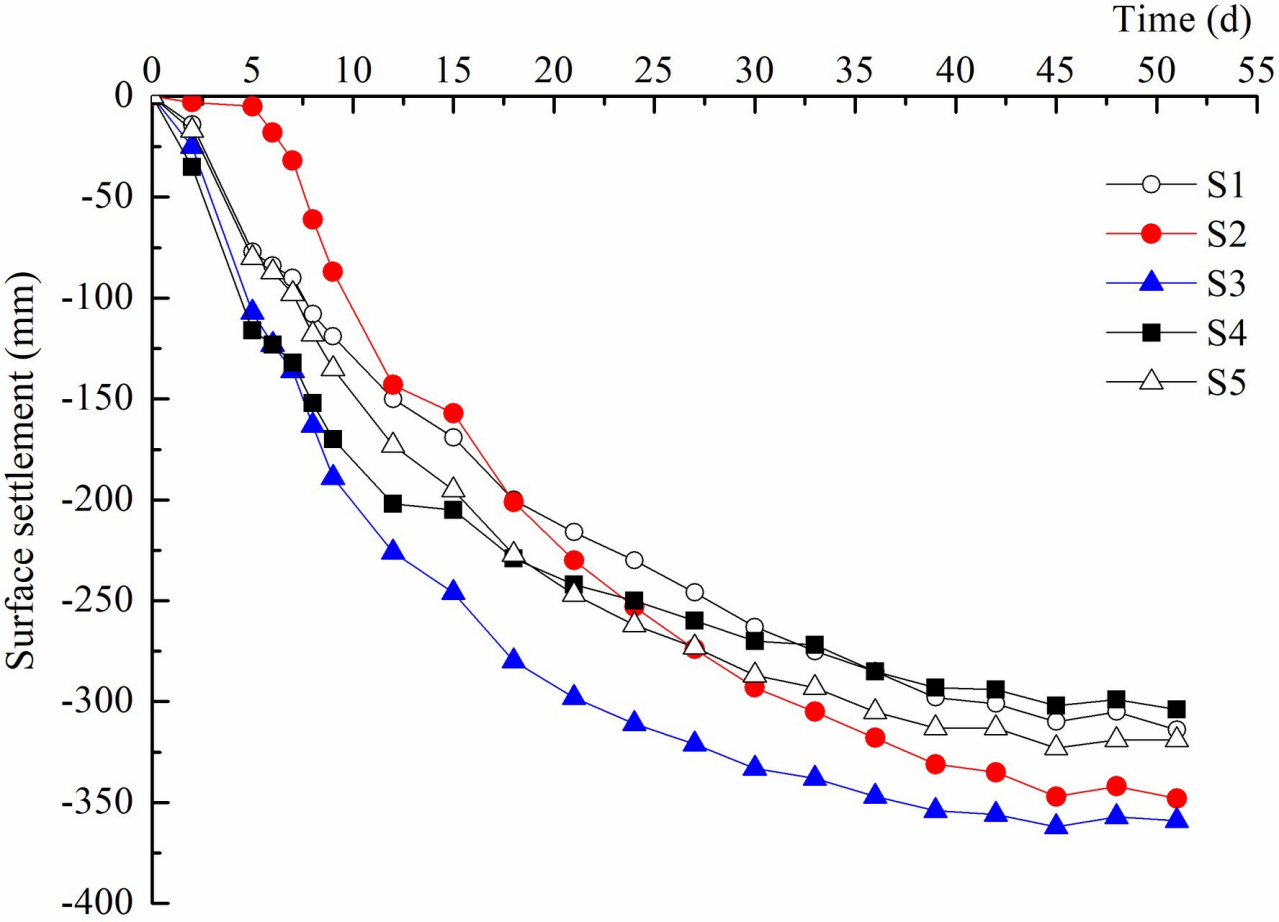
Surface settlement variation.

The surface settlements of the five settlement plates are shown in Fig 7. Before the 15 d of reinforcement there was a rapid, almost linear increase in the surface settlements. Then the settlement rate decreased gradually and tended to be steady after 45 d of reinforcement. The maximum and minimum final settlement was 35.9 cm for S3 located on the southeast side of the site and 30.4 cm for S4 located on the southern side of the site, respectively. It is necessary to judge whether the consolidation degree is suitable for excavation according to the final excavation situation. The degree of consolidation *U*_*t*_ can reflect the reinforcement effect and dissipation of excess pore water pressure most directly, which is expressed as follows:

$$U_{t}=\frac{S_{t}}{S_{\infty }}$$ (1)

where *S*_*t*_ is the average settlement at time *t*, and $S_{\infty }=\frac{S_{3}\left(S_{2}-S_{1}\right)-S_{2}\left(S_{3}-S_{2}\right)}{\left(S_{2}-S_{1}\right)-\left(S_{3}-S_{2}\right)}$ is the final settlement of foundation [22]. *S*_1_, *S*_2_ and *S*_3_ are the settlements at time intervals *t*_1_, *t*_2_ and *t*_3_, respectively. The time intervals should be long enough and try to make *t*_3_ at the end of the settlement curve. After repeated selection and calculation the final degrees of consolidation for S1-S5 were 83.3%, 82.7%, 85.7%, 84.0%, and 81.6%, respectively.

There are mainly two reasons contributing to the generation of the settlement. Soil is a three-phase dispersion system. The discharge of pore water and air into the soil, and the movement of fine soil particles resulting in realign of soil particles, both caused the decrease of soil pore volume and hence the compression of soil under the effect of vacuum preloading and direct-current electric field. The suitable compression of the soil was conducted to reduce the mass and volume of the excavated soil.

### Analysis of soil laboratory testing

**Fig 8:**
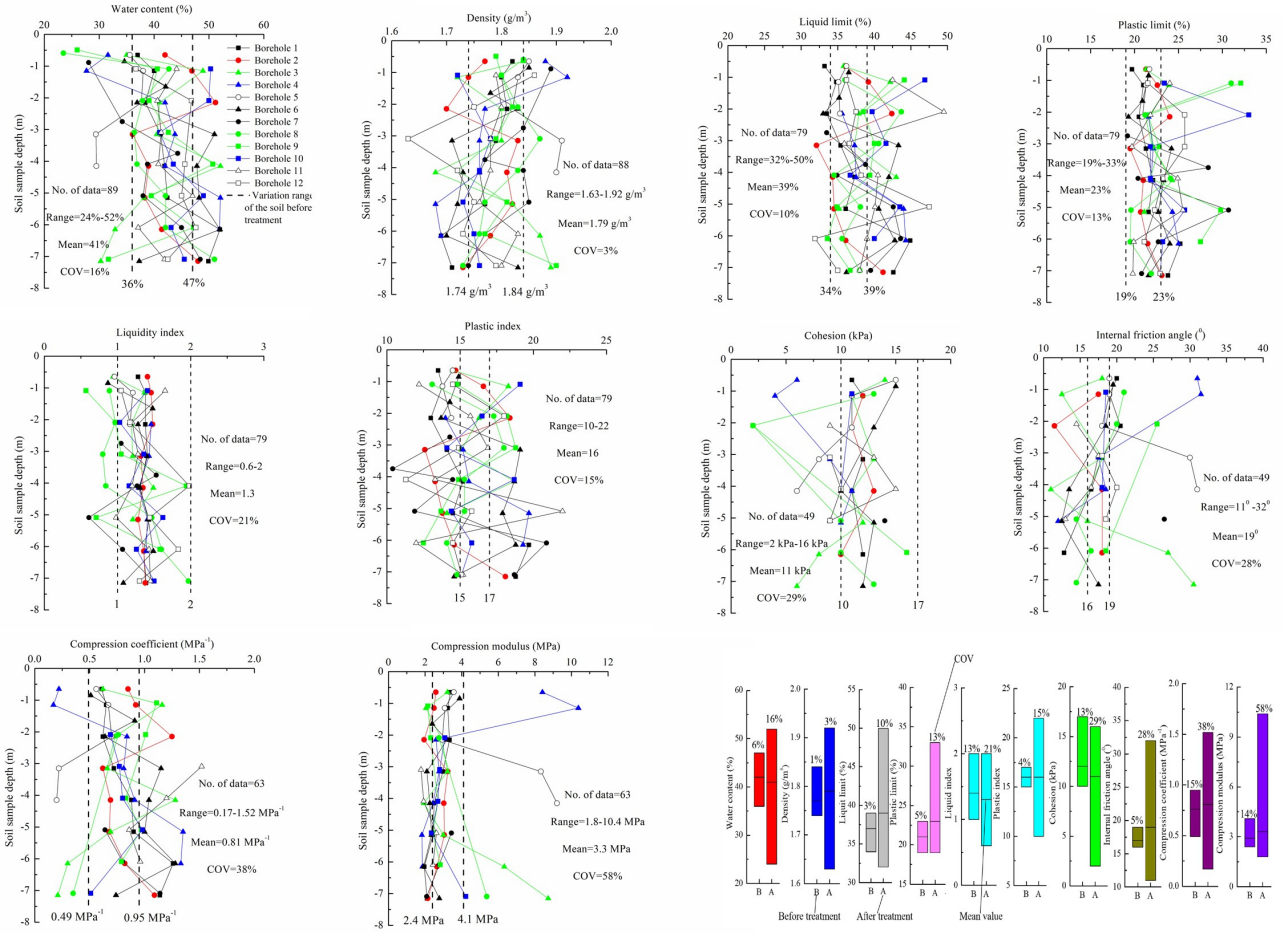
Soil parameter distribution along its depth profile of different boreholes. a) Water content. b) Density. c) Liquid limit. d) Plastic limit. e) Liquidity index. f) Plastic index. g) Cohesion. h) Internal friction angle. i) Compression coefficient. j) Compression modulus. k) Variation range, mean value, and COV of the soil parameters before and after treatment.

Twelve 7 m deep boreholes were drilled in the construction site after VPE treatment for soil laboratory testing. The vertical distribution of soil physical, mechanical and deformation parameters are shown in Fig 8A–8J, respectively. Fig 8K using bar charts illustrates the variation range, mean value, and COV of the soil parameters before and after treatment. Firstly, the variations of the physical parameters after VPE treatment were analyzed. As seen from the laboratory test soil samples for most of the boreholes the water content increased and the density decreased along the depth direction. The soil water contents after VPE treatment ranged from 24% to 52%, while most of the water contents was still between the range of 36% and 47% (before treatment range). About 16% water content data was smaller than the low end of the initial soil water content range. The water content of soil within the depth of 1 m decreased most significantly. The mean value of the soil water content before and after VPE treatment was close, the COV increased from 6% to 16%. The soil density varies from 1.63 to 1.92 g/cm^3^ after VPE treatment exceeded the previous range before treatment. The testing locations of the density data larger than the initial upper limit were coincidently almost consistent with that of water content smaller than the initial lower limit of water content. The testing locations of the density data smaller than the lower limit were coincidently almost consistent with that of water content larger than the initial upper limit of water content. The COV and mean of density after treatment was slightly larger than that before treatment (S4 and S5 Datasets).

As shown in Fig 8C and 8D, there are obviously some data larger than the initial upper limit for the liquid limit and plastic limit. Only very little data is slightly below the low end of liquid limit before treatment range, while no data below that of plastic limit before treatment range. If the soil plastic limit exceeded the initial upper limit, its corresponding liquid limit at the same location also almost exceeded its initial upper limit. The COV and mean value for liquid limit after VPE treatment increased by 7% and 2%, respectively. Similarly, the COV and mean value for plastic limit after VPE treatment increased by 8% and 2%, respectively. Basically, the liquid limit and plastic limit of the soil at some locations increased after VPE treatment. Liquid limit is an important index property for cohesive soils. It reflects the particle-level interactions and soil microstructure. The increase in liquid limit indicates further fundamental changes in the clay properties, which may be due to the increase of salt content of the soil after VPE treatment [28, 29]. The presence of gel generated by electroosmosis sorbed the extra amount of water by matrix suction might result in an increase in the plastic limit [30] (S6 and S7 Datasets).

For liquidity index, it was larger than 1 for the soil before treatment, while after VPE treatment 11% of testing data ranged from 0.75 to 1 representing the soil changing from a fluid state to a soft plastic state and 4% of data ranged from 0.25 to 0.75 representing the soil changing from a fluid state to a plastic state. Its mean value decreased by 0.1 and COV increased by 8% more than the soil before treatment. For plastic index, the mean value after treatment was the same as that before treatment. However, it ranged from 10 to 22, with 51% data smaller than the initial lower limit and 28% data larger than the initial upper limit. The COV also increased by 11% (S8 and S9 Datasets).

Secondly, the variations of the mechanical parameters after VPE treatment were analyzed. For cohesion, there is some data smaller than the initial lower limit, the mean value decreased by 1 kPa. For internal friction angle, it ranged from 11° to 32° and its mean value was 19° increasing by 2° than the initial mean value. There is 29% of the data larger than the initial upper limit. Most of the testing locations of the internal friction angles larger than the initial upper limit were coinciding with that of cohesion data smaller than the lower limit of the soil cohesion before treatment. Conversely, the corresponding internal friction angles of the cohesion data smaller than the lower limit of the soil cohesion before treatment were larger than the initial upper limit of internal friction angles before treatment (S10 and S11 Datasets).

Finally, the variations of the deformation parameters after VPE treatment were analyzed. There is 11% compression coefficient data smaller than 0.5 MPa^-1^ and its corresponding compression modulus larger than 4 MPa. But there is 25% compression coefficient data larger than 1 MPa^-1^. Although the mean compression coefficient and the mean compression modulus increased by 0.05 MPa^-1^ and 0.4 MPa respectively compared to the initial values, their COV increased by 23% and 44%, respectively (S12 Dataset).

Although the mean values of the soil index parameters after VPE treatment improved slightly, their COV significantly increased, which was attributed to the inhomogeneity strengthening effect of VPE for different dewatering amounts of electroosmosis flow between the anodes and the cathodes in the horizontal direction and the decay of vacuum pressure and voltage potential in the vertical direction [26]. The COV of soil parameters after VPE treatment all increased compared to that before treatment. This indicated that after VPE treatment the dispersion of soil parameters became larger. The COV of deformation parameters were larger than that of mechanical parameters, and the latter were larger than that of physical parameters. Inversely, the stability of physical parameters is better than the mechanical parameters, and the latter is better than the deformation parameters after VPE treatment.

In addition, water content is an easily available index of soil properties. With water content as an independent variable, linear regression was used to fit the correlation among indexes, hence simplifying the calculation and acquisition of soil physical property indexes. It is found that water content (*w*) has a good linear relationship with density (*ρ*) and compression coefficient (*a*_1‒2_). The linear fitting equations are as follows:

$$\rho =-0.0092w+2.1673\left(R^{2}=0.9854\right)$$ (2)

$$a_{1-2}=0.0457w-1.1013\left(R^{2}=0.9502\right)$$ (3)

## Foundation pit excavation

**Fig 9:**
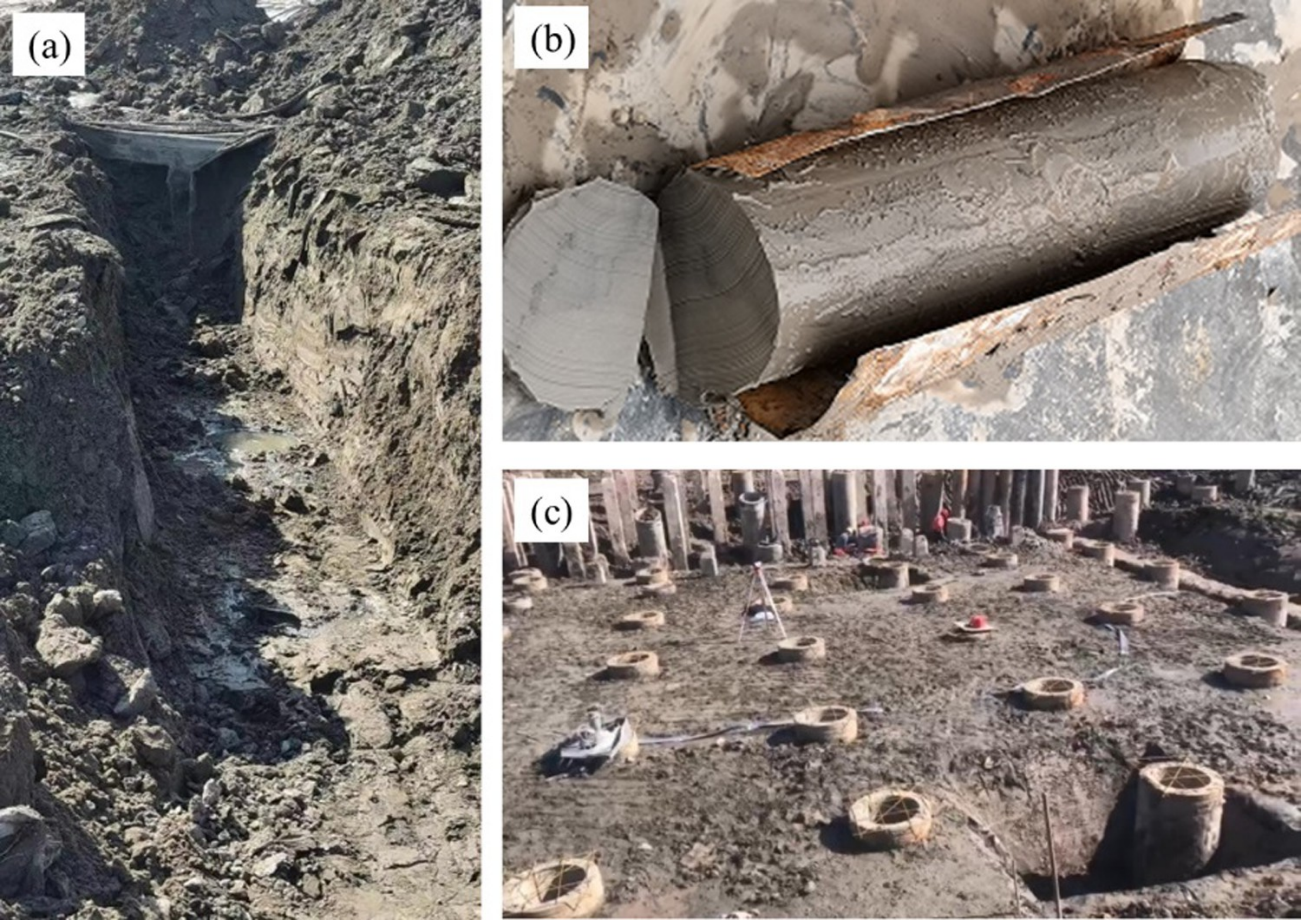
Soil state after treatment. a) Miniature excavation. b) Drill hole sample at 5 m. c) Scene after completion of excavation.

From the laboratory testing, the results indicated that the soil properties after VPE treatment distribution had greater spatial variability. Although there was little difference between the mean values of soil parameters before and after treatment, the miniature excavation soil was hard and the pit wall was stable (Fig 9A), the drill hole sample had good continuity and integrity with stable forming (Fig 9B). This indicated that the improved index parameters scattering in the construction site have a significant influence on the soil sate. After comprehensive judgement, it has been proven that the treated soft soil can carry out foundation pit excavation. The practice has proved that the subsequent construction tasks such as pit slope and excavation have been successfully realized in this area as shown in Fig 9C, and the construction of the sluice project has been carried out.

## Conclusions

A sluice foundation pit excavation project located at tidal flats site experienced well point precipitation and tube well drainage. However, unstable sliding occured during the slope excavation of the foundation pit and caused the failure of some pre-driven piles in the local scope of the site. VPE technique was selected for engineering application specifically for the disturbed soil with complex terrain after comprehensive analysis of the construction craft, scope of application, and technical and economical characteristics of the further disposal schemes.

The electrification mode significantly influenced electric energy consumption. Stepped-up voltage helped save electric energy, but electrode conversion consumed a lot of electric energy. When the consolidation degree reached above 80%, it was suitable for further excavation. The dispersion of soil parameters became larger after VPE treatment. The stability of physical parameters is better than the mechanical parameters, and the latter is better than the deformation parameters. Soil water content has a linear relationship with density and compression coefficient. The given linear fitting equations can help in simplifying the calculation and acquisition of these indexes. Although the mean values of the soil index parameters slightly improved, their COV significantly increased. These locations with improved index parameters scattering in the construction site ensured that the subsequent construction tasks such as pit slope and excavation were successfully realized in this area. VPE method can be applied in treating soft soil during foundation pit excavation project. The successful excavation of this project showed that the mean values of the soil index parameters after VPE treatment changed slightly making it less important in judging the likelihood of excavation of the soil, while the values with great improvement emphasized the favorable excavation.

## Supporting information

S1 Dataset Electric current. (OPJ)

S2 Dataset Electric energy consumption. (OPJ)

S3 Dataset Pore water pressure. (OPJ)

S4 Dataset Water content. (OPJ)

S5 Dataset Density. (OPJ)

S6 Dataset Liquid limit. (OPJ)

S7 Dataset Plastic limit. (OPJ)

S8 Dataset Liquidity index. (OPJ)

S9 Dataset Plastic index. (OPJ)

S10 Dataset Cohesion. (OPJ)

S11 Dataset Internal friction angle. (OPJ)

S12 Dataset Compression modulus. (OPJ)

## References

[1] Lin P, Chen X, Jiang M, Song X, Xu M, Huang S. Mapping shear strength and compressibility of soft soils with artificial neural networks. Eng Geol. 2022; 300: 106585. 10.1016/j.enggeo.2022.106585

[2] Onyelowe K, Van DB, Igboayaka C, Orji F, Ugwuanyi H. Rheology of mechanical properties of soft soil and stabilization protocols in the developing countries-Nigeria. Materials Science for Energy Technologies. 2019; 2(1): 8–14. 10.1016/j.mset.2018.10.001

[3] Chai J, Carter JP. Simulation of the progressive failure of an embankment on soft soil. Comput Geotech. 2009; 36(6): 1024–1038. 10.1016/j.compgeo.2009.03.010

[4] Kong G, Fang J, Lv Z, Yang Q. Effects of pile and soil properties on thermally induced mechanical responses of energy piles. Comput Geotech. 2023; 154: 105176. 10.1016/j.compgeo.2022.105176

[5] Zhou Y, Kong G, Li J. Performance of a belled pile influenced by pile head freedom response to a cooling–heating cycle. J Geotech Geoenviron. 2023; 149(2): 04022133. 10.1061/JGGEFK.GTENG-10407

[6] Huang M, Liu X, Zhang N, Shen Q. Calculation of foundation pit deformation caused by deep excavation considering influence of loading and unloading. J Cent South Univ. 2017; 24(9): 2164–2171. 10.1007/s11771-017-3625-3

[7] Davies RV. Some geotechnical problems with foundations and basements in Singapore. Proc Conf On Tall Buildings. 1984; 643–650.

[8] Karlsrud K, Andresen L. Loads on braced excavations in soft clay. Int J Geomech. 2005; 5(2): 107–113. 10.1061/(ASCE)1532-3641(2005)5:2(107)

[9] Fan J, Wang D, Qian D. Soil-cement mixture properties and design considerations for reinforced excavation. J Rock Mech Geotech Eng. 2018; 10(4): 791–797. 10.1016/j.jrmge.2018.03.004

[10] Onur MI, Tuncan M, Evirgen B, Ozdemir B, Tuncan A. Behavior of soil reinforcements in slopes. Procedia engineering. 2016; 143: 483–489. 10.1016/j.proeng.2016.06.061

[11] Oh BS. A Case study on the cause and reinforcement of railroad facilities settlement according to the ground excavation. Journal of the Korean GEO-environmental Society. 2012; 13(10): 85–94.

[12] Nikbakhtan B, Osanloo M. Effect of grout pressure and grout flow on soil physical and mechanical properties in jet grouting operations. Int J Rock Mech Min. 2009; 46(3): 498–505. 10.1016/j.ijrmms.2008.10.005

[13] Ignat R, Baker S, Larsson S, Liedberg S. Two-and three-dimensional analyses of excavation support with rows of dry deep mixing columns. Comput Geotech. 2015; 66: 16–30. 10.1016/j.compgeo.2015.01.011

[14] Jiang W. Dewatering of the deep excavation for a high rise building. Soil Engineering and Foundation. 2015; 293: 34.

[15] Andromalos KB, Hegazy YA, Jasperse BH. Stabilization of soft soils by soil mixing. Soft ground technology. 2001; 194–205. 10.1061/40552(301)16

[16] Hu ZF, Yue ZQ, Zhou J, Tham LG. Design and construction of a deep excavation in soft soils adjacent to the Shanghai Metro tunnels. Can Geotech J. 2003; 40(5): 933–948. 10.1139/t03-041

[17] Nidheesh PV, Kumar MS. An overview of environmental sustainability in cement and steel production. J Clean Prod. 2019; 231: 856–871. 10.1016/j.jclepro.2019.05.251

[18] Minjun SHI. Optimization design and application of support system in foundation pit in soft soil area. Soil Engineering and Foundation. 2021; 35(3): 286.

[19] Wu YX, Shen SL, Xu YS, Sun WJ. Groundwater control during deep excavation in the soft deposit of China: An Overview. International Conference on Ground Improvement & Ground Control. 2012; 10.3850/978-981-07-3560-9_07-0702

[20] Gouw TL. Case histories on the application of vacuum preloading and geosynthetic-reinforced soil structures in Indonesia. Indian Geotech J. 2020; 50: 213–237. 10.1007/s40098-019-00391-5

[21] Pujades E, Vàzquez-Suñé E, Carrera J, Jurado A. Dewatering of a deep excavation undertaken in a layered soil. Eng Geol. 2014; 178: 15–27. 10.1016/j.enggeo.2014.06.007

[22] Liu F, Fu H, Zhou J, Wang J, Cai Y, Zhao R, et al. Vacuum preloading combined with intermittent electro-osmosis for dredged slurry strengthening. Geotech Test J. 2019; 43(3): 775–790. 10.1520/GTJ20180234

[23] Wang J, Fu H, Liu F, Cai Y, Zhou J. Influence of electro-osmosis activation time on vacuum electro-osmosis consolidation of a dredged slurry. Can Geotech J. 2018; 55(1): 147–153. 10.1139/cgj-2016-0687

[24] Sun Z, Gao M, Yu X. Vacuum preloading combined with electro-osmotic dewatering of dredger fill using electric vertical drains. Dry Technol. 2015; 33(7): 847–853. 10.1080/07373937.2014.992529

[25] López-Acosta NP, Espinosa-Santiago AL, Pineda-Núñez VM, Ossa A, Mendoza MJ, Ovando-Shelley E, et al. Performance of a test embankment on very soft clayey soil improved with drain-to-drain vacuum preloading technology. Geotext Geomembr. 2019; 47(5): 618–631. 10.1016/j.geotexmem.2019.103459

[26] Sun Z, Lu L, Gong J, Wei G, Ye W. Shear strength performance of electrokinetic geosynthetics treated soft clay after water immersion. Processes. 2023; 11(2): 529. 10.3390/pr11020529

[27] Saowapakpiboon J, Bergado DT, Chai JC, Kovittayanon N, de Zwart TP. Vacuum-PVD combination with embankment loading consolidation in soft Bangkok clay: a case study of the Suvarnabhumi Airport Projec. Geosynthetics in Civil and Environmental Engineering: Geosynthetics Asia 2008 Proceedings of the 4th Asian Regional Conference on Geosynthetics in Shanghai, China. Springer Berlin Heidelberg. 2009; 440–449.

[28] Morris DV, Hillis SF, Caldwell JA. Improvement of sensitive silty clay by electroosmosis. Can Geotech J. 1985; 22(1): 17–24. 10.1139/t85-003

[29] Lo KY, Ho KS. The effects of electroosmotic field treatment on the soil properties of a soft sensitive clay. Can Geotech J. 1991; 28(6): 763–770. 10.1139/t91-093

[30] Emerson WW. The plastic limit of silty, surface soils in relation to their content of polysaccharide gel. Soil Res. 1995; 33(1): 1–9. 10.1071/SR9950001

